# Disability among adults with diagnosed HIV in the United States, 2017

**DOI:** 10.1080/09540121.2020.1842318

**Published:** 2020-11-10

**Authors:** Pranesh P. Chowdhury, Linda Beer, Fengjue Shu, Jennifer Fagan, R. Luke Shouse

**Affiliations:** a Division of HIV/AIDS Prevention, CDC, Atlanta, GA, USA; b ICF International, Atlanta, GA, USA

**Keywords:** Disability, depression, Smoking, HIV infection

## Abstract

In the United States, one in four adults is living with a disability. Age-related changes, disease-related pathology and treatments can place a person with HIV at risk for a disability. We analyzed nationally representative data to describe disability status among adults ≥18 years with diagnosed HIV in the United States and Puerto Rico by demographic characteristics, health behaviors, quality of care, clinical outcomes and mental health status. We reported weighted percentages and prevalence ratios with predicted marginal means to evaluate significant differences between groups (*P* < .05). Overall, 44.5% reported any disability; the most frequently reported disabilities were related to mobility (24.8%) and cognition (23.9%). Persons who lived in households at or below the poverty level or who experienced homelessness in the last 12 months reported a higher prevalence of any disability than persons who were not poor or not homeless (60.2% vs. 33.4% and 61.8% vs. 42.8%, respectively). Prevalence of depression and anxiety was higher among persons with any disability compared with those with no disability (32.8% and 26.6% versus 10.1% and 7.0%, respectively). Enhancing support from clinicians and ancillary providers may help optimize long-term health outcomes among HIV-positive persons with disabilities.

## Introduction

The Centers for Disease Control and Prevention (CDC) defines a disability as a condition that makes it more difficult for a person to do certain activities and interact with the world around them. In the United States, 61 million adults are living with some type of disability (Okoro et al., 2018) and the three most frequently reported disabilities were related to mobility (13.7%), cognition (10.8%) and independent living (6.8%). Prevalence of disability increases with age (Courtney-Long et al., 2015). With the advances in antiretroviral therapy (ART) treatments, people with HIV are living longer (Antiretroviral Therapy Cohort Collaboration, 2008). Persons with HIV infection may be more likely to have disabilities because nearly half of the people with HIV are over 50 years (CDC, 2018) and many older adults with HIV experience health-related deterioration from aging (Pathai et al., 2014), HIV-related comorbidities (Leveille & Thapa, 2017), as well as negative long-term effects of HIV treatments (Onen & Overton, 2011). It is important to know how disability can affect people with HIV and understanding who is disproportionately affected by disability may be helpful for public health prevention. The purpose of this analysis was to use Medical Monitoring Project (MMP) data to describe the prevalence of disability status overall and by selected characteristics, among adults with diagnosed HIV living in the United States and Puerto Rico.

## Materials and methods

MMP is a surveillance system that produces nationally representative estimates of behavioral and clinical characteristics of adults aged ≥18 years with diagnosed HIV living in the United States and Puerto Rico. We used interview and medical record data collected from 4222 adults living with HIV collected from June 2017 through May 2018. Details about MMP sampling, data collection and weighting processes were described previously (Beer et al., 2019). All analyses were conducted using SAS callable SUDAAN version 11.03 (RTI International, Research Triangle Park, NC) to account for the complex survey design and weights. We estimated the weighted prevalence and 95% confidence interval (CI) of reporting at least one disability overall and by variables capturing socio-demographics, any Ryan White HIV/AIDS Program (RWHAP) assistance, unmet needs, quality of care, clinical outcomes, health behaviors and mental health. To compare groups, unadjusted prevalence ratios (PR) with CIs were calculated using logistic regression with predicted marginal means (Bieler et al., 2010).

MMP included six questions about disabilities related to hearing, vision, cognition, mobility, self-care and independent living (HHS, 2011). Respondents were asked “Are you deaf or do you have serious difficulty hearing?” (hearing disability); “Are you blind or do you have serious difficulty seeing, even when wearing glasses?” (vision disability); “Because of a physical, mental, or emotional condition, do you have serious difficulty concentrating, remembering, or making decisions?” (cognition disability); “Do you have serious difficulty walking or climbing stairs?” (mobility disability); “Do you have difficulty dressing or bathing?” (self-care disability) and “Because of a physical, mental or emotional condition, do you have difficulty doing errands alone such as visiting a doctor’s office or shopping?” (independent living disability). Respondents could report more than one disability. Persons who responded “yes” to at least one of these questions were identified as having any disability and those who responded “no” to all six questions were identified as having no disability. Socio-demographics, RWHAP assistance, unmet needs, mental health, adherence to ART, and health behaviors were self-reported for the past 12 months unless otherwise indicated. Mental health status included symptoms of depression or anxiety in the past two weeks prior to the interview. ART prescription and viral suppression measures were abstracted from medical records.

## Results

Overall, 44.5% (CI 42.7–46.4) of adults with diagnosed HIV reported any disability; 13% reported three or more disabilities. The most frequently reported disability was difficulty related to mobility (24.8%, CI 23.2–26.4), followed by cognition (23.9%, CI 22.2–25.6), independent living (12.5%, CI 11.3–13.8), vision (12.5%, CI 11.0–14.1), hearing (9.7%, CI 8.5–11.1) and self-care (7.0%, CI 6.1–8.1) (not in tables). The prevalence of any disability differed across MMP project areas, ranging from 33.6% in Georgia to 57.0% in Puerto Rico (Table 1).

Women reported a higher prevalence of any disability than men (53.6% vs. 41.3%) (Table 2). Among age groups, the prevalence of any disability was highest among adults aged 65 years or older (60.4%) and lowest among those aged 18–24 years (32.2%). Among racial/ethnic groups, Hispanic/Latino adults reported the highest prevalence of any disability (48.9%). Persons who lived in households at or below the poverty level were 80% (PR = 1.80, CI 1.65–1.96) and who were homeless in the last 12 months were 44% (PR = 1.44, CI 1.31–1.59) more likely to report any disability than persons who were not poor or not homeless. Persons who went without food due to lack of money or who had at least one unmet ancillary service need were 76% (PR = 1.76, CI 1.65–1.89) and 66% (PR = 1.66, CI 1.51–1.81) more likely to report any disability than their counterparts, respectively.

There was no association between disability status and ART prescription, adherence to HIV medications, and viral suppression (Table 3). Persons with any disability were 34% (PR = 1.34, CI 1.24–1.46) as likely to be current smoker than person with no disability. Similarly, compared with persons who did not have a disability, person with disability were over three times (PR = 3.27, CI 2.87–3.27) as likely to report depression and nearly four times (PR = 3.81, CI 3.12–4.65) as likely to report anxiety. When we stratified the analysis by number of disabilities (data not presented in table), compared with persons who did not have a disability, persons with three or more disabilities were 10% (PR = 0.90, CI 0.83–0.98) less likely to be adherent to HIV medicine, 57% (PR = 1.57, CI 1.40–1.75) more likely to be a current smoker, over four times (PR = 4.36, CI 3.73–5.11) as likely to report depression, and over five times (PR = 5.31, CI 4.08–6.91) as likely to report anxiety. There was no association between number of disabilities and ART prescription or viral suppression.

## Discussion

The prevalence of any disability among adults with diagnosed HIV is higher than in the general population (44.5% vs. 25.7%) and is also higher among those aged 45–64 years (49.6% vs. 28.6%) and 65 or more years (60.4% vs. 41.7%) – indicating the substantial burden of disability among persons with HIV (Okoro et al., 2018). Like previous studies among the general population (Courtney-Long et al., 2015), the wide variation in the prevalence of disabilities among adults with diagnosed HIV across U.S. jurisdictions may reflect geographic differences in demographic factors, health behaviors, health care access or combinations of these factors, and highlights the importance of monitoring of disability status in this population by area.

With advances in efficacy and tolerability of ART, people diagnosed with HIV are living longer; as the number of older persons living with HIV increases (Pathai et al., 2014), the prevalence of disabilities may also increase. Similar to what has been found among the general population (Okoro et al., 2018), disabilities related to difficulties with mobility and cognition were the most frequently reported. The CDC-recommended self-management intervention, “Living Well with a Disability” (Ravesloot et al., 2016), may be an effective tool to improve the health and quality of life among adults with HIV who are living with disabilities.

Our findings indicate that adults with HIV who are living with any disability may need enhanced access to ancillary services that can support their needs for housing, food or nutrition, substance abuse and mental health services to improve their health outcomes (Conviser & Pounds, 2002). We did not find any difference in RWHAP assistance between persons with and without disability, although persons with a disability may have a higher need for services available through Ryan White. The federally funded Ryan White HIV/AIDS Program provides access to medical and support services for nearly half a million persons living with HIV in the USA (Kaiser, 2019) and expanding its patient-centered medical home model may improve access to care for persons with disabilities (Pappas et al., 2014).

The findings of this analysis are subject to limitations. First, self-reported information may be subject to biases that may lead to measurement error. Second, disability measures were self-reported and are not official designations for any Social Security benefit. However, self-report is the most commonly used method to assess disability for surveillance purposes. A strength of this analysis was the use of the Department of Health and Human Service’s standard self-reported six-question measure of any disability, which facilitates monitoring of disability status among people with HIV and enables comparisons of disability prevalence across different population-based studies. Third, we cannot assess causality due to MMP’s cross-sectional design.

Successful treatment and management of HIV infection have transformed HIV into a chronic disease. However, optimizing health outcomes among persons with HIV with a disability may require enhanced support from clinicians and ancillary providers.

## Figures and Tables

**Table 1.** Prevalence of any disability among U.S. adults with diagnosed HIV by area – Medical Monitoring Project, 2017 (*N* = 4222).

**MMP project areas**	**Total**	**Any disability**		
	*N* *	*n* *	%^†^	95% CI^§^

National	4202	1886	44.5	(42.7–46.4)
Puerto Rico	183	105	57.0	(48.5–65.1)
New York-State	424	212	50.2	(44.6–55.8)
New York City	349	177	51.3	(44.7–57.8)
Illinois-State	253	120	48.1	(40.9–55.4)
Chicago	176	83	42.0	(33.8–50.6)
Washington	183	83	47.3	(39.7–55.1)
Mississippi	139	70	46.8	(36.4–57.5)
Delaware	186	88	46.4	(39.3–53.7)
California-State	582	271	45.6	(40.6–50.6)
Los Angeles County	171	91	50.8	(42.0–59.5)
San Francisco	185	74	40.7	(33.7–48.1)
Michigan	180	82	45.6	(37.6–53.8)
North Carolina	179	80	45.5	(38.1–53.1)
Indiana	173	77	44.8	(36.9–53.0)
Florida	289	126	43.7	(37.2–50.5)
Oregon	218	95	43.6	(34.9–52.6)
New Jersey	228	98	43.3	(36.0–50.9)
Pennsylvania-State	251	105	41.0	(33.7–48.6)
Philadelphia	165	70	42.5	(33.0–52.7)
Texas-State	358	140	40.7	(34.9–46.8)
Houston	173	63	35.8	(28.3–43.9)
Virginia	165	63	37.5	(29.5–46.3)
Georgia	211	71	33.6	(27.4–40.5)

CI, confidence interval.

* Numbers are unweighted.

† Percentages are weighted row percentages.

§ CIs incorporate weighted percentages.

**Table 2.** Prevalence of any disability among U.S. adults with diagnosed HIV by selected characteristics – Medical Monitoring Project, 2017 (*N* = 4222).

**Participant characteristics**	**Total**	**Any disability**			**Prevalence ratio** (95% CI)	***p*-value of *t*-test *β* = 0**
	*N* *	*n* *	%^†^	95% CI^§^		

Total	4202	1886	44.5	(42.7–46.4)	-	-
**Gender**						
Female	1035	574	53.6	(49.8–57.4)	1.30 (1.21–1.40)	<0.001
Male	3096	1270	41.3	(39.5–43.1)	Referent	-
**Self-identified sexual orientation**						
Lesbian or gay	1801	642	35.8	(33.4–38.2)	Referent	-
Heterosexual or Straight	1923	1006	51.2	(48.5–53.9)	1.43 (1.33–1.53)	<0.001
Bisexual	363	177	48.8	(42.4–55.2)	1.36 (1.16–1.60)	<0.001
Other^¶^	86	43	48.0	(37.2–59.0)	1.34 (1.05–1.71)	0.030
**Age at time of interview (years)**						
18–24 years	92	29	32.2	(21.9–44.6)	Referent	-
25–44 years	1366	451	32.9	(30.2–35.7)	1.02 (0.70–1.50)	0.911
45–64 years	2400	1203	49.6	(47.5–51.8)	1.54 (1.07–2.23)	0.010
65 or more	344	203	60.4	(53.8–66.6)	1.88 (1.25–2.83)	<0.001
**Race/Ethnicity**						
White non-Hispanic	1200	480	41.7	(38.6–45.0)	0.93 (0.80–1.07)	0.316
Black non-Hispanic	1717	773	44.1	(41.4–46.8)	0.98 (0.86–1.11)	0.742
Hispanic/Latino**	954	479	48.9	(44.7–53.1)	1.09 (0.93–1.27)	0.309
Other^††^	331	154	45.0	(39.3–50.9)	Referent	-
**Education status**						
Less than high school	690	452	63.4	(58.9–67.6)	1.67 (1.51–1.85)	<0.001
High school diploma or GED	1113	525	46.5	(43.8–49.3)	1.22 (1.12–1.33)	<0.001
More than high school	2395	906	38.0	(35.4–40.6)	Referent	-
**Employment status**						
Employed^§§^	1992	527	26.3	(24.2–28.5)	Referent	-
Not employed	1762	1155	65.4	(63.0–67.7)	2.49 (2.29–2.70)	<0.001
Other^¶¶^	441	201	44.8	(39.9–49.9)	1.71 (1.49–1.95)	<0.001
**Household income at or below poverty level in the past 12 months** ***						
Yes	1660	1008	60.2	(57.3–62.9)	1.80 (1.65–1.96)	<0.001
No	2263	752	33.4	(31.1–35.8)	Referent	-
**Time since HIV diagnosis**						
<5 years	658	219	33.2	(29.3–37.4)	Referent	-
5–9 years	865	353	42.2	(38.7–45.6)	1.27 (1.08–1.49)	0.003
10 or more years	2679	1314	48.1	(45.4–50.8)	1.45 (1.27–1.65)	<0.001
**Homelessness at any time in the past 12 months** ^†††^						
Yes	390	252	61.8	(56.7–66.7)	1.44 (1.31–1.59)	<0.001
No	3812	1634	42.8	(40.9–44.8)	Referent	-
**Any Ryan White HIV/AIDS Program (RWHAP) assistance, past 12 months**						
Yes	1985	911	45.7	(43.5–47.9)	1.06 (0.98–1.14)	0.149
No	2114	918	43.2	(40.7–45.7)	Referent	-
**Needed or used SSI** ^§§§^ **in the past 12 months**						
Yes	782	527	66.1	(62.4–69.7)	1.68 (1.57–1.80)	<0.001
No	3380	1328	39.3	(37.4–41.3)	Referent	-
**Needed or used SSDI** ^§§§^ **in the past 12 months**						
Yes	948	661	68.6	(65.7–71.4)	1.83 (1.72–1.94)	<0.001
No	3216	1199	37.5	(35.5–39.5)	Referent	-
**Went without food due to lack of money in the past 12 months**						
Yes	863	582	67.6	(64.5–70.6)	1.76 (1.65–1.89)	<0.001
No	3338	1304	38.4	(36.5–40.4)	Referent	-
**At least one unmet ancillary service needs in the past 12 months** ^¶¶¶^						
Yes	2191	1197	54.7	(52.0–57.4)	1.66 (1.51–1.81)	<0.001
No	1998	682	33.0	(30.6–35.5)	Referent	-

CI, confidence interval; GED, general educational development; SSI, supplemental security income; SSDI, social security disability insurance.

* Numbers are unweighted.

† Percentages are weighted row percentages.

§ CIs incorporate weighted percentages.

¶ Includes something else.

** Hispanics or Latinos might be of any race. Persons are classified in only 1 race/ethnicity category.

†† Includes American Indian/Alaska Native, Asian, Native Hawaiian/Other Pacific Islander, and multiple races.

§§ Employed includes employed for wages, self-employed, or homemaker.

¶¶ Includes a student and retired.

*** Poverty guidelines as defined by HHS: https://aspe.hhs.gov/frequently-asked-questions-related-poverty-guidelines-and-poverty.

††† Living on the street, in a shelter, in a single-room–occupancy hotel, or in a car.

§§§ Disabilities are self-reported and are not official designations for any Social Security benefits.

¶¶¶ Unmet need was defined as an ancillary service that the participant reported as needed but not received during the 12 months before the interview. Ancillary services for which unmet need was assessed were: HIV case management, adherence counseling, AIDS Drug Assistance Program, patient navigator, HIV peer group support, dental health, drug or alcohol counseling, mental health, transportation assistance, SSI, SSDI, food assistance, meals or food services, domestic violence, interpreter, and lawyer or legal.

**Table 3.** Clinical outcomes, health behaviors and mental health status of U.S. adults with diagnosed HIV by disability status – Medical Monitoring Project, 2017 (*N* = 4222).

	**Any disability**			**No disability**			**Prevalence ratio**^¶^ (95% CI)	***p*-value of *t*-test *β* = 0**
	*n* *	%^†^	95% CI	*n*	%	95% CI		

**Clinical Outcomes**								
Prescribed ART in the past 12 months**	1684	85.8	(83.1–88.1)	2040	83.0	(80.4–85.2)	1.03 (1.00–1.07)	0.077
100% adherent to HIV medicine in the past 30 days^††^	1039	59.1	(56.6–61.6)	1355	62.2	(60.1–64.2)	0.95 (0.90–1.00)	0.048
Most recent viral load documented undetectable or <200 copies/mL in the past 12 months	1394	69.9	(66.0–73.0)	1780	70.1	(65.5–74.3)	1.00 (0.95–1.05)	0.896
All viral load measurements documented undetectable or <200 copies/mL in the past 12 months	1253	63.3	(59.7–66.8)	1598	63.2	(59.0–67.2)	1.00 (0.94–1.07)	0.944
**Health behaviors**								
Current Smoker	719	39.1	(35.7–42.5)	640	29.1	(26.8–31.5)	1.34 (1.24–1.46)	<0.001
Binge drinking in the past 30 days^§§^	260	13.7	(12.0–15.7)	413	17.0	(15.1–19.0)	0.81 (0.70–0.94)	0.004
Heavy drinking in the past 30 days^¶¶^	95	4.8	(3.9–5.9)	135	5.4	(4.5–6.6)	0.89 (0.65–1.21)	0.447
Any drug use in the past 12 months	590	31.8	(28.8–35.0)	713	29.6	(26.9–32.6)	1.07 (0.96–1.20)	0.202
**Mental health**								
Had major or other depression (based on DSM-IV)^***^ during the two weeks before the interview	607	32.8	(29.8–36.0)	222	10.1	(8.9–11.4)	3.27 (2.87–3.72)	<0.001
Had anxiety based on GAD 7^†††^ scale during the two weeks before the interview	501	26.6	(24.1–29.3)	162	7.0	(5.9–8.3)	3.81 (3.12–4.65)	<0.001

CI, confidence interval; ART, antiretroviral therapy.

* Numbers are unweighted.

† Percentages are weighted row percentages.

§ CIs incorporate weighted percentages.

¶ Comparing people with no disability vs. who had any disability.

** ART prescription documented in medical record; persons with no medical record abstraction were considered to have no documentation of ART prescription.

†† Did not miss a single dosage of any HIV medicines in the past 30 days.

§§ Defined as drinking ≥5 alcoholic beverages in a single sitting (≥4 for women) during the 30 days before the interview.

¶¶ Defined as consuming an average of >2 drinks/day or >14 drinks/week (for men) or consuming an average >1 drink/day or >7 drinks/week (for women) during the 30 days before the interview.

*** Responses to the items on the PHQ-8 were used to define "major depression” and "other depression”, according to criteria from the DSM-IV. "Major depression” was defined as having at least five symptoms of depression; "other depression” was defined as having two to four symptoms of depression.

††† Responses to the Generalized Anxiety Disorder Scale (GAD-7) were used to define anxiety according to criteria from DSM-IV. Anxiety was defined as having a score of ≥10.
